# Specific Eccentric–Isokinetic Cluster Training Improves Static Strength Elements on Rings for Elite Gymnasts

**DOI:** 10.3390/ijerph16224571

**Published:** 2019-11-18

**Authors:** Christoph Schärer, Lisa Tacchelli, Beat Göpfert, Micah Gross, Fabian Lüthy, Wolfgang Taube, Klaus Hübner

**Affiliations:** 1 Department of Elite Sport, Swiss Federal Institute of Sport Magglingen (SFISM), 2532 Magglingen, BE, Switzerland; micah.gross@baspo.admin.ch (M.G.); fabian.luethy@baspo.admin.ch (F.L.); klaus.huebner@baspo.admin.ch (K.H.); 2 Department of Neurosciences and Movement Sciences, University of Fribourg, 1700 Fribourg, Switzerland; lisa.tacchelli@bluewin.ch (L.T.); wolfgang.taube@unifr.ch (W.T.); 3 Department Biomedical Engineering (DBE), University of Basel, 4123 Allschwil, BL, Switzerland; beat.goepfert@unibas.ch

**Keywords:** strength training, eccentric, isokinetic, upper limbs, artistic gymnastics, rings, males

## Abstract

In gymnastics, coaches are constantly searching for efficient training methods in order to improve the athletes’ performance. Therefore, in this study we aimed to investigate the effects of a novel, four-week, gymnastic-specific, eccentric–isokinetic (0.1 m/s) cluster training on a computer-controlled training device on the improvement of two static strength elements on rings (swallow and support scale). Nine elite male gymnasts participated in this study. Outcome parameters were maximum strength and strength endurance in maintaining the static position of both elements. After four weeks of training, specific maximum strength increased significantly (swallow: +4.1%; *d* = 0.85; *p* = 0.01; support scale: +3.6%; *d* = 2.47; *p* = 0.0002) and strength endurance tended to improve (swallow: +104.8%; *d* = 0.60; *p* = 0.07; support scale: +26.8%; *d* = 0.27; *p* = 0.19). Our results demonstrate that top athletes can considerably improve ring-specific strength and strength endurance in only four weeks. We assumed that the high specificity but also the unfamiliar stimulus of slow eccentric movements with very long times under maximal muscle tension led to these improvements. We suggest to use this type of training periodically and during phases in which the technical training load is low.

## 1. Introduction

Elite athletes have to train in a very specific way in order to achieve optimal performance. In this context, the optimization of specific physical prerequisites, such as strength, power, or muscular endurance, is indispensable in many sports [1]. At the same time, athletes face the problem of overuse injuries due to the high overall volume of their sport-specific and preparatory (e.g., resistance) training. In gymnastics, where the acquisition of technical skills is the main aim of the training, very high training loads have been previously reported [2]. In this context, it was shown that top female gymnasts train up to 40 h per week and perform up to 400,000 elements (gymnastics skills that are assigned to a value in the code of points [3]) per year [2]. Together with the improvement of technique and the acquisition of more difficult skills, physical prerequisites must be developed simultaneously. Therefore, coaches and athletes are constantly looking for even more efficient preparatory exercises that allow the athletes to improve their sport-specific performance in a short period of time. For this reason, male artistic gymnastics coaches recently added specific eccentric exercises on rings (e.g., lowering from handstand to swallow on rings) to maximize the specific maximum strength. 

**Figure 1:**
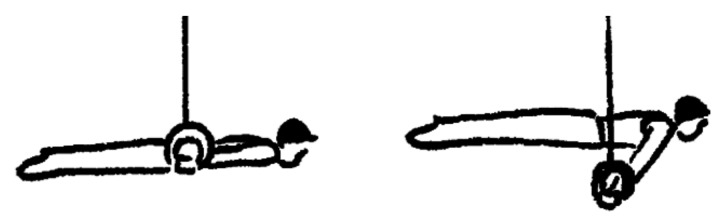
Swallow and support scale. Perfect execution of the swallow (**left**) and support scale (**right**) strength hold elements on rings, which must be maintained for 2 s according to the code of points [3].

Maintaining a static strength element on rings may be at least partly considered an eccentric muscle contraction due to the decelerating muscle work required in order to overcome gravity while maintaining the static positions. Consequently, the adjunct of ring-specific eccentric exercises in gymnastics may be useful. Further, a high level of relative maximum strength of the upper limbs and advanced balance skills in the hold positions are crucial to be able to perform these elements [4,5,6]. In order to perform a maximum number of strength elements in a competition routine (up to eight are allowed), a high level of specific strength endurance is required. Within the existing strength elements on rings in the code of points [3], the swallow and support scale are important hold elements in order to achieve a high difficulty score in competitions (Figure 1).

Eccentric training is a highly efficient method for increasing maximum strength. In particular, it is a low-energy-cost method to overload the muscles [7] and may be advisable for sports with high loads and subtle coordination [8]. Eccentric exercises are generally executed with high loads that exceed the maximal concentric force of the muscles, resulting in a stretching of the muscle while it contracts [9]. The overload and the eccentric contraction provokes muscle damage and delayed onset muscle soreness [10], but results in longer-term improved maximal strength [11] and muscle coordination [8]. Furthermore, in order to develop the same force for a given external load [12], fewer motor units are recruited with eccentric compared to concentric contractions. As a result, eccentric exercises provoke a higher mechanical stress per motor unit than concentric training methods [13]. 

Despite the possible benefits of performing eccentric strength exercises on rings, these may pose a considerable injury risk. The rings are an unstable apparatus, and therefore gymnasts need to constantly control the position of the rings so that they do not lose their balance during the lowering of the body. Together with the high joint torque due to the acceleration of the eccentric movement under “isoinertial” (constant load) conditions [14], ring-specific eccentric exercises may provoke torque on the joints of the upper limbs that is hard to control without damaging the muscle tendon structures. 

Coaches are convinced of the effectiveness of eccentric training for increasing the specific strength of hold elements on rings, but the applied methods and exercises on rings pose a risk of injury or may cause shoulder pain. Further, it is very difficult to control the load of these traditional isoinertial eccentric exercises. Therefore, we intended to implement a novel and effective strength training method for elite gymnasts to boost their specific strength for the swallow and support scale elements by means of a computer-controlled device. By using very slow isokinetic execution for an exercise in the supine position that involves similar muscle groups as when performing the swallow and support scale strength elements, we removed the two factors (instability of the rings and acceleration under isoinertial conditions) that are difficult to control in the traditional ring-specific eccentric training.

Hence, the aim of this study was to investigate the effects of a four-week training intervention with this specific eccentric–isokinetic training method on maximal strength and strength endurance for the swallow and support scale elements. 

## 2. Materials and Methods

### 2.1. Subjects

Nine international or national top-level gymnasts (members of the national team) (age: 21.47 ± 1.96 y; height: 169.84 ± 5.47 cm; weight: 69.4 ± 7.0 kg) volunteered to participate in this study. All athletes trained professionally (weekly training volume: 24 h) and were free of injury. They were informed of the benefits and risks of the investigation prior to signing an informed consent to participate in the study. The measurements were approved by the Ethics Committee of Bern (Project-ID: 2017-01891) and conducted in accordance with the current version of the Declaration of Helsinki, the guidelines for good clinical practice (ICH-GCP ISO EN 14,155), and all national legal and regulatory requirements.

### 2.2. Procedures

**Figure 2:**
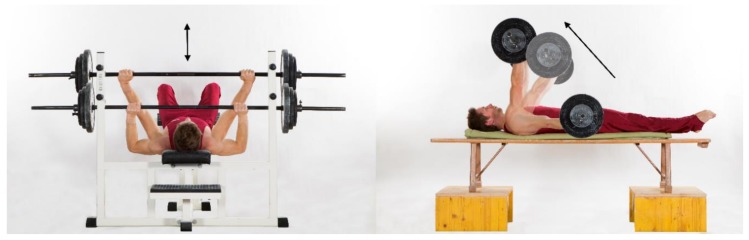
Preconditioning strengthening exercises. Execution of the one-repetition maximum test for the bench press (**left**) and swallow supine position (**right**) exercises.

The athletes completed a four-week training intervention, including two ring-specific eccentric–isokinetic training sessions per week. The tests and intervention took place in an early preparatory phase for competitions with rather low but similar training load for all of the participants. One week before (pre-test), as well as one (one-week post-test) and three weeks (three-week post-test) after the intervention, three specific maximal strength tests took place to test the acute and delayed effects (transformation to sport-specific performance) of the applied strength training [15]. In order to ensure recovery, the day before the tests the gymnasts only trained in the morning and had a half-day off in the afternoon. For each test, the gymnasts warmed-up individually (general gymnastics warm-up) and then first performed a one-repetition-maximum test (1RM) of the preconditioning strengthening exercises, namely the bench press and “swallow supine position”, according to Hübner and Schärer (Figure 2) [16]. Both exercises were executed in a supine position. For bench press, the barbell was lowered until it touched the gymnast’s chest and then pushed up until the arms were straight. The swallow supine position started with straight arms and the hands positioned on the bar one hand-width wider than the shoulders. Then, with fully extended arms, the barbell was lifted until arms were vertical (90 degrees), while maintaining contact between the bench and the back and head at all times. The starting weight was chosen individually by the gymnasts (approximately 90% of the 1RM) and increased by 2.5 kg after each successful attempt until the athletes failed to lift it. Athletes had a five-minute rest between attempts.

**Figure 3:**
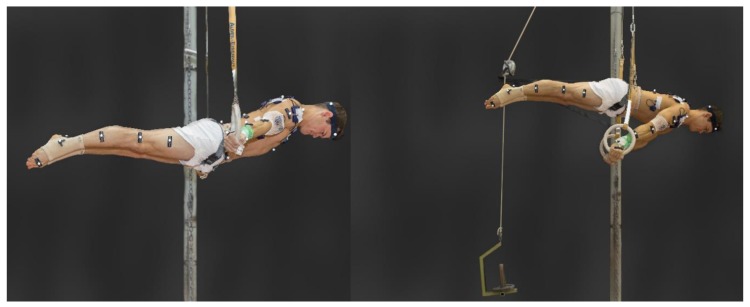
Test conditions for static exercises. The swallow (**left**) and support scale (**right**) exercises were performed with maximum resistance for five seconds. These examples show the use of additional weight (**left**) and the pulley and counterweight (**right**).

Next, the maximum strength for the swallow and support scale exercises was assessed on the rings. For this, either a pulley-system with a counterweight (resistance less than body mass) or a weight belt (resistance greater than body mass) were used (Figure 3) to determine the maximal strength (body mass + additional weight from weight belt or counterweight) when maintaining the exercises for five seconds [6]. Finally, in order to assess changes in strength endurance at one-week and three-week post-test, athletes performed a maximal duration hold of both elements using the maximum load obtained (over 5 s) in the pre-test. For each hold, time measurement started when the stable hold position was reached during at least two subsequent video frames and stopped when the athlete departed from the hold position. After each tested exercise, the athletes had at least five minutes of recovery.

Trials were only valid if the angular deviation of the performed elements was smaller than 45°, in accordance with the requirements for recognition of strength elements in the international gymnastics federation’s code of points [3]. 

In order to monitor the execution of the performed elements, trials were captured with nine 3D-cameras (Vicon Vantage, Vicon Motion Systems Ltd., Oxford, UK), operating at 120 Hz and placed around the rings in an upper (height: 4.6 m) and lower plane (height: 0.4–1.4 m). Forty-three reflective markers (14 mm diameter) were placed on the gymnasts’ lower and upper limbs, according to the plug-in-gait model [17]. Angular deviations from the prescribed perfect hold positions were analyzed using modeled markers (ProCalc, Vicon Motion Systems Ltd., UK) midway between the two proximal wrist markers, the two shoulder markers, the two ankle markers, and the four hip markers, resulting in a two-dimensional stick figure, which displayed the relevant body angles. All trials that were included in the study met the abovementioned requirements for recognition (5 s holding time, angular deviations within the requirements). Further, the execution of both swallow and support scale elements was statistically similar (mean deviation of relevant body angles from the perfect position: 8.56° ± 7.66°) across all tests (one-way ANOVA with repeated measures: *p* > 0.05).

### 2.3. Eccentric–Isokinetic Training

**Figure 4:**
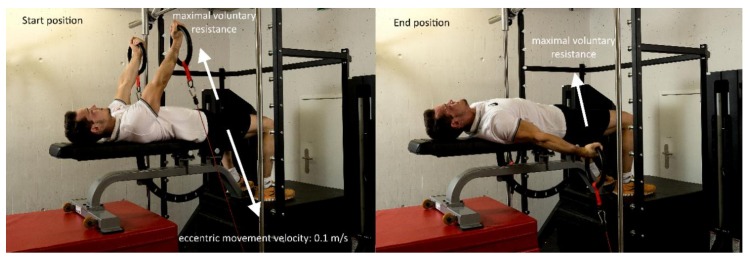
Training exercise. Start (**left**) and end positions (**right**) of the eccentric–isokinetic training exercise for the swallow and support scale elements performed on the 1080 Quantum Syncro (1080 Motion, Lidingö, Sweden). The movement velocity of 0.1 m/s yielded a mean eccentric duration of ~5 s per repetition.

The four-week eccentric–isokinetic training was performed on a computer-controlled training device (1080 Quantum Syncro, 1080 Motion, Lidingö, Sweden). Despite the unfamiliar training setting, the intention was to create a training exercise that was as similar as possible to the execution of the swallow and support scale elements. To this end, gymnasts lay supine on a bench with arms outstretched holding a set of rings, which were fixed to synchronized cables. As seen in Figure 4, the start position was with a shoulder angle of 50–70° (corresponding to that while performing the support scale) and the end position was at a shoulder angle of −15° to −30° (corresponding to the maximum range of motion while performing the swallow). During the execution of each eccentric–isokinetic contraction, the cables were reeled in synchronously with a constant velocity of 0.1 m/s, while athletes provided maximal voluntary resistance with fully extended arms. The very slow eccentric velocity of 0.1 m/s was chosen in order to simulate the muscle work while holding a strength element on rings (maximal and nearly isometric contraction with decelerating muscle work). Further, the mean duration of one repetition at the chosen velocity was 4.81 ± 1.18 s, which corresponds to a typical holding duration for strength elements during training on rings. In order to limit rest between repetitions, athletes had to return to the starting position as fast as possible after each repetition.

During the intervention, the number of sets and total repetitions per session varied from week to week, with an overall increase across the intervention (Table 1). Sets were separated by five minutes of recovery and were performed as 3–4 clusters of four repetitions each, where clusters were separated by brief rest periods of 20 s. The aim of the rest periods between clusters was to maximize the number of repetitions while maintaining the highest possible quality (applied maximum force) for each repetition [18]. In addition, the few repetitions per cluster would allow the athletes to maximize applied force throughout the set.

In order to ensure complete recovery between the training sessions, athletes did not perform strength elements on rings during the intervention. Further, to ensure a controlled and standardized training protocol, athletes were asked to avoid other maximum strength training exercises for the upper extremities from pre-test to three-week post-test. The technical training load aside from the intervention (24 h per week) was similar for all participating athletes.

Ten days before the first training session, athletes were familiarized with the training device and the eccentric–isokinetic exercise by performing one set with four clusters of four repetitions each. The familiarization session was intended to serve as practice in the correct execution of the exercise and in order to limit muscle soreness (common following unfamiliar eccentric exercise) during the four-week training intervention (repeated bout effect) [19,20].

### 2.4. Statistical Analyses

Descriptive statistics were performed on all variables. Normal distribution of the data was confirmed with Shapiro–Wilk test. One-way analysis of variance (ANOVA) with repeated measures was used to calculate overall effects. T-tests (post-hoc) and effect sizes (Cohen’s d) were used to assess changes between the three tests. Effect sizes were classified according to Cohen [21] as small (0.2–0.49), medium (0.5–0.79), or large (≥0.8). The level of statistical significance was set to *p* < 0.05. P-values were adjusted using the Holm-Bonferroni correction [22]. All calculations were performed using SPSS 22 software (SPSS, Inc., Chicago, IL, USA).

## 3. Results

Due to the absence of one athlete (training camp abroad), only eight athletes were present at three-weeks post-test. Further, three gymnasts did not perform the preconditioning bench press exercise in any of the tests, and one athlete did not perform the third 1RM swallow supine position test due to shoulder pain (only when performing this exercise) unrelated to investigated training intervention. Where an athlete did not perform an exercise at all three time points, the data from that exercise were excluded from analysis.

During the four weeks of training, body mass did not change significantly. One-way ANOVA of the preconditioning exercises revealed a strong tendency towards improvement for swallow supine position (*d* = 0.58; *p* = 0.06), but a smaller effect for bench press (*d* = 0.37; *p* = 0.28). Post-hoc tests showed tendencies (large effects) towards improvement for both exercises (Table 2).

### 3.1. Maximum Strength

**Figure 5:**
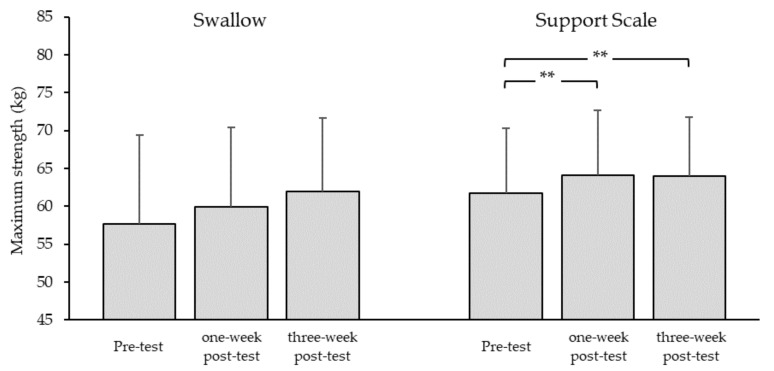
Maximum strength. Mean values and standard deviations for maximal strength (body mass—counterweight or body mass + additional weight) for the swallow and support scale elements when held for 5 s on rings (*n* = 8). Pre-test = before training; one-week post-test and three-week post-test = one and three weeks after the four-week eccentric–isokinetic training intervention, respectively; ** significant change: *p* < 0.01.

One-way ANOVA showed large effects and significant improvements in maximum strength for swallow (*d* = 0.85; *p* = 0.01) and support scale (*d* = 2.47; *p* = 0.0002). Post-hoc tests (*t*-tests) revealed a tendency towards improvement of maximum strength for swallow (+4.1%) from pre-test to three-week post-test (*d* = 0.98; *p* = 0.08) and a medium effect size between pre-test and one-week post-test (*d* = 0.69; *p* = 0.14). Further, maximum strength for the support scale exercise increased significantly and with a large effect size from pre-test to one-week post-test (+3.6%; *d* = 1.76; *p* = 0.06), and from pre-test to three-week post-test (*d* = 1.50; *p* = 0.08; Figure 5).

### 3.2. Strength Endurance

**Figure 6:**
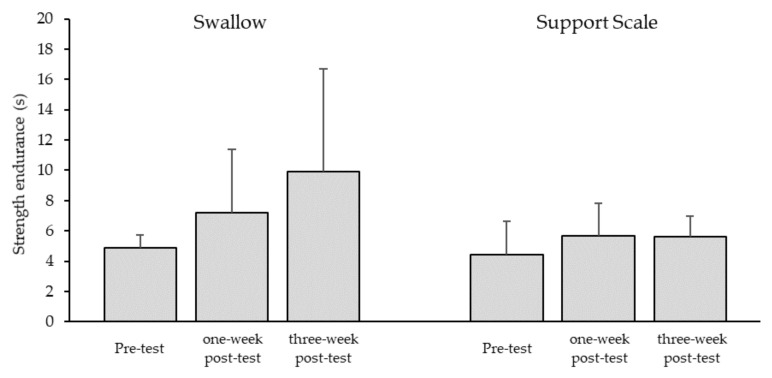
Strength endurance. Mean values and standard deviations for the maximum holding time, with the maximum resistance attained in pre-test (reference) for the swallow and support scale elements on rings (*n* = 8). Pre-test = before training; one-week and three-week post-test = one and three weeks after the four-week eccentric–isokinetic training, respectively.

One-way ANOVA revealed a tendency towards improvement in strength endurance for the swallow element (*d* = 0.60; *p* = 0.07) and a small effect for support scale (*d* = 0.27; *p* = 0.19). Post-hoc analysis showed that the increase in strength endurance for the swallow element *(+ 104.8*% from pre- to three-week post-test) was non-significant and of medium effect size between all tests (pre-test vs. one-week post-test: *p* = 0.20, *d* = 0.59; pre-test vs. three-week post-test: *p* = 0.20; *d* = 0.75; one-week post-test vs. three-week post-test: *p* = 0.20, *d* = 0.77). However, a large effect on strength endurance for support scale was found between pre-test and three-week post-test *(+26.3*%; *p* = 0.12, *d* = 0.88), and a medium effect between pre-test and one-week post-test (*p* = 0.37, *d* = 0.52) was found, whereas no change occurred between one-week and three-week post-test (*p* = 0.98, *d* = 0.01) (Figure 6).

## 4. Discussion

To the best of our knowledge, this is the first study to analyze the effects of a four-week, ring-specific, eccentric–isokinetic training intervention on maximum strength and strength endurance for the swallow and support scale elements in male elite gymnasts. Despite the fact that the athletes were highly trained and competed at an international or national level, the four-week eccentric–isokinetic cluster training was able to further improve their performance. Specifically, athletes demonstrated significantly improved maximum strength and a tendency towards improvement in strength endurance for the swallow and support scale elements following training. Furthermore, 1RM for the bench press and “swallow supine position” preconditioning strengthening exercises tended to improve. 

Concerning the effects of eccentric strength training, no general consensus can be found in the scientific literature. Most studies show positive effects of eccentric training on strength or performance, but some authors have questioned the generalizability of its effects for well-trained athletes [8]. Further, greater maximum strength gains have been attributed to eccentric–isoinertial (constant load) training than to eccentric–isokinetic (constant velocity) training [8,23]. While these previous studies mostly investigated the effect of rather unspecific training exercises on highly specific sport performance, we intended to create a training exercise that was as similar as possible to the sport-specific performance, yet in a highly controlled setting. Therefore, the ring-specific adaptations may have been strongly dependent on the similarity between the demands of the trained movement and the static strength elements on rings. Furthermore, the slow isokinetic execution of the training exercise may have evoked similar muscle activation as during a static hold element, due to the decelerating muscle work while maintaining the static position in order to overcome gravity. Moreover, the slightly smaller adaptations in the (concentric) 1RM for the swallow supine position and bench press preconditioning exercises support the conclusion of Vogt and Hoppeler [9] that the magnitude of strength gains with eccentric training is higher when tested eccentrically than concentrically.

In general, our results indicate that eccentric (in our case, isokinetic) training can improve the performance of even highly trained elite athletes. One prerequisite for this may be that the movement and the movement velocity of the applied exercise is similar to the sport-specific performance. This may be an important factor for coaches (and scientists) if they intend to implement novel, efficient “preparatory” exercises to improve athletes’ performance.

In men’s artistic gymnastics, ring-specific strength is traditionally trained by either (a) concentric barbell exercises [16,24], (b) holding the static position of the elements with the help of the coach, or (c) eccentric–isoinertial exercises (e.g., lowering from handstand to swallow). In this context, it should be mentioned that all of the traditional training methods have different advantages and disadvantages. Barbell exercises are rather unspecific (unspecific movements and concentric muscle work), but may be indispensable to improve the maximum strength in the upper extremities. Performing static strength elements on rings with the help of the coach may be important to improve the balance skills in the static position, but due to the dependence on the coach’s help, it is difficult to control the intensity of the performed exercise. Eccentric exercises on rings may be very specific but the instability of the rings and the acceleration of the isoinertial condition may provoke excessive torque on the shoulder joints, thus posing a risk for injury. The athletes who participated in the current study had employed each of the aforementioned training methods repeatedly over the prior years. Since exposing athletes to a variety of stimuli has been shown to be crucial for ensuring a continuous improvement in maximal strength [1], it could be that the very well-trained athletes in this study were so accustomed to the traditional training methods that they would have been unable to experience further improvements in specific maximum strength using these methods. On the other hand, the eccentric–isokinetic method employed in this study represented a novel and unfamiliar yet specific training stimulus for the athletes, and this could be the reason for the improvements of the ring-specific strength the athletes experienced within such a short period of time. One example to illustrate this is the performance of athlete 9 (see Table S1). The holding position for the swallow element of this athlete changed considerably after the four week of training. At pre-test he performed the swallow with slightly bent arms (execution error). Probably due to the training exercise that was executed with completely straight arms, he could only perform the element with perfectly straight arms (without execution error) at the one- and three-week post-tests. This incidental improvement in holding technique made it difficult for him to hold the swallow element with the maximum resistance (body mass + counterweight) of the pre-test (resulting in 0 s holding time for strength endurance at one- and three-week post-test). A few months later, after having gotten used to this new and better technique, he was able to perform the swallow element with perfectly straight elbows at competitions. In order to prevent injuries and due to the high load on the shoulder joints while performing static strength elements on rings, these elements are usually maintained for a total only 30 to 60 s at most in one training session (e.g., 3 series of 3 × 5 s). The time under maximal tension per strength training session in this study (2–4 min per training) was up to four times greater than usual holding times per training with other methods. This may be another reason for the observed improvements in maximal strength and strength endurance in the static holding positions. Improved ring-specific endurance may help the gymnasts to increase the number of strength elements performed in their routines on rings, and therefore increase their chances of success in competitions.

In elite sports and especially in individual sports, it is difficult to recruit an adequate number of athletes for studies. Further, all elite athletes want to benefit from new training methods, and therefore in intervention studies with elite athletes, control groups are very rare. Hence, these studies are limited to describe the effects of the applied method, and cannot compare the effectiveness of different methods. However, the “few” athletes that participated in our study were not only national team members but some of them were even medalists at European Championships. Therefore, the results of our study may be considered representative, even for top-level gymnasts.

## 5. Conclusions

The four-week eccentric–isokinetic training intervention employed in the current study increased the specific (static) maximal strength and revealed a tendency towards improvement in strength endurance for the swallow and support scale elements of elite male gymnasts, probably due to the fact that it was a new and highly specific stimulus (regarding the involved muscle groups, slow eccentric movement, long time under maximal muscle tension) for these well-trained athletes. Nevertheless, the high physical strain on the upper limbs from the eccentric–isokinetic intervention could have acute negative effects on the athletes’ performance in their regular (technical) training on the apparatus. For this reason, and in order to prevent injuries, the volume of technical training during this type of eccentric–isokinetic strength training should be prescribed according to the fatigue of the athletes. Further, we suggest that this type of training should be used periodically, not continuously, for example during an early preparatory phase in which the technical training load is usually lower.

## Figures and Tables

**Table 1:** Eccentric–isokinetic training protocol. Sets, clusters, repetitions (reps), rest duration, and time under tension (mean duration per rep ~ 5 s) for the four-week eccentric–isokinetic training for the swallow and support scale elements.

Week	Trainings Per Week	Sets–Clusters–Reps Per Training (Rest Duration)	Time under Tension Per Training (Per Week)
1	2	2–4–4 (5 min–20 s–none)	~ 2 min 40 s (5 min 20 s)
2	2	3–3–4 (5 min–20 s–none)	~ 3 min (6 min)
3	2	2–3–4 (5 min–20 s–none)	~ 2 min (4 min)
4	2	3–4–4 (5 min–20 s–none)	~ 4 min (8 min)

**Table 2:** Body mass and preconditioning exercises. Mean values ± standard deviations (SD) of the body mass and the one-repetition-maximum (1RM) for the preconditioning exercises bench press and swallow supine position (swallow sup) before (Pre) and one and three weeks after (1-w-post and 3-w-post) the 4-week eccentric–isokinetic training, as well as effect sizes (Cohen’s d) and significance of changes (*p*-values from *t*-tests).

	*n*	Mean *± SD* (kg)	Pre vs. 1-w-Post d *(p)*	Pre vs. 3-w-Post d *(p)*	1-w Post vs. 3-w-Post d *(p)*
*Body mass*					
Pre	8	68.6 ± *7.1*	0.21 *(0.58)*	0.03 *(0.93)*	0.14 *(0.70)*
1-w-post	8	68.5 *± 6.8*			
3-w-post	8	68.6 *± 7.3*			
*Bench press (1RM)*					
Pre	5	105.0 *± 16.6*	0.29 *(0.55)*	0.73 *(0.36)*	1.10 *(0.21)*
1-w-post	5	106.5 *± 19.1*			
3-w-post	5	108.0 *± 18.5*			
*Swallow sup (1RM)*					
Pre	7	43.9 *± 10.0*	0.75 *(0.23)*	0.80 *(0.23)*	0.21 *(0.60)*
1-w-post	7	45.7 *± 10.6*			
3-w-post	7	46.1 *± 10.7*			

## Supplementary Materials

The following are available online at https://www.mdpi.com/1660-4601/16/22/4571/s1. Table S1: Raw data of the study.
